# Uncoupling protein 3 deficiency impairs myocardial fatty acid oxidation and contractile recovery following ischemia/reperfusion

**DOI:** 10.1007/s00395-018-0707-9

**Published:** 2018-10-29

**Authors:** Kristin S. Edwards, Sadia Ashraf, Tyler M. Lomax, Jessica M. Wiseman, Michael E. Hall, Fabio N. Gava, John E. Hall, Jonathan P. Hosler, Romain Harmancey

**Affiliations:** 1 Department of Physiology and Biophysics, University of Mississippi Medical Center, 2500 N. State St., Jackson, MS 39216-4505 USA; 2 Mississippi Center for Obesity Research, University of Mississippi Medical Center, Jackson, MS USA; 3 Mississippi Center for Heart Research, University of Mississippi Medical Center, Jackson, MS USA; 4 Department of Medicine, University of Mississippi Medical Center, Jackson, MS USA; 5 Department of Cell and Molecular Biology, University of Mississippi Medical Center, Jackson, MS USA

**Keywords:** Type 2 diabetes, Myocardial infarction, Uncoupling protein, Cardiac metabolism, Mitochondrial function

## Abstract

Patients with insulin resistance and type 2 diabetes have poor cardiac outcomes following myocardial infarction (MI). The mitochondrial uncoupling protein 3 (UCP3) is down-regulated in the heart with insulin resistance. We hypothesized that decreased UCP3 levels contribute to poor cardiac recovery following ischemia/reperfusion (I/R). After confirming that myocardial UCP3 levels were systematically decreased by 20–49% in animal models of insulin resistance and type 2 diabetes, we genetically engineered Sprague–Dawley rats with partial loss of UCP3 (ucp3^+/−^). Wild-type littermates (ucp3^+/+^) were used as controls. Isolated working hearts from ucp3^+/−^ rats were characterized by impaired recovery of cardiac power and decreased long-chain fatty acid (LCFA) oxidation following I/R. Mitochondria isolated from ucp3^+/−^ hearts subjected to I/R in vivo displayed increased reactive oxygen species (ROS) generation and decreased respiratory complex I activity. Supplying ucp3^+/−^ cardiac mitochondria with the medium-chain fatty acid (MCFA) octanoate slowed electron transport through the respiratory chain and reduced ROS generation. This was accompanied by improvement of cardiac LCFA oxidation and recovery of contractile function post ischemia. In conclusion, we demonstrated that normal cardiac UCP3 levels are essential to recovery of LCFA oxidation, mitochondrial respiratory capacity, and contractile function following I/R. These results reveal a potential mechanism for the poor prognosis of type 2 diabetic patients following MI and expose MCFA supplementation as a feasible metabolic intervention to improve recovery of these patients at reperfusion.

**Electronic supplementary material:**

The online version of this article (10.1007/s00395-018-0707-9) contains supplementary material, which is available to authorized users.

## Introduction

In the United States alone, more than 35 million individuals are suffering from diabetes mellitus. By 2030, this diabetes epidemic is expected to extend to more than 54 million Americans and will lead to nearly 400,000 deaths annually [57]. Cardiovascular disease is the most prevalent cause of mortality in diabetic patients [43]. Type 2 diabetes mellitus represents > 95% of all diabetes cases [24]. The hyperinsulinemia and reduced insulin sensitivity which characterize this metabolic disorder are linked to a two- to fourfold increase in the risk for ischemic heart disease [2, 25]. Consequently, diabetic individuals represent a rapidly growing number of the patients undergoing reperfusion for acute myocardial infarction [63]. In addition to increasing the incidence of myocardial infarction (MI), diabetes is also associated with increased cardiac morbidity and mortality following revascularization [15, 19, 66]. While the pathophysiology of cardiovascular diseases in diabetes is complex and multifactorial, specific molecular mechanisms may underlie the poor prognosis of the diabetic patient population in response to ischemia/reperfusion (I/R).

Uncoupling protein 3 (UCP3) is a mitochondrial anion carrier protein with antioxidant properties known to stimulate long-chain fatty acid (LCFA) metabolism in muscle cells [46, 60]. In prediabetic subjects and type 2 diabetic patients, skeletal muscle UCP3 content is decreased by almost 50% [59]. Regulation of myocardial UCP3 with hyperinsulinemia and type 2 diabetes is less clear with reports of either unchanged [12, 34], increased [51], or decreased expression [11, 32, 33], in the rodent models explored so far. In mice, we previously demonstrated that chronic hyperinsulinemia down-regulates myocardial UCP3 content by 40% through induction of pathway-selective insulin resistance, increased recruitment of the sterol regulatory element-binding protein (SREBP)-1 transcription factor at the UCP3 promoter, and transcriptional repression of the UCP3 gene [31]. In rats rendered insulin resistant by high-sucrose feeding, we observed a similar decrease in cardiac UCP3 levels that was associated with decreased rates of myocardial LCFA oxidation after ischemia and impaired recovery of contractile function [33]. In mice, genetic deletion of UCP3 has been associated with decreased myocardial ATP content at reperfusion [53], as well as worsening of cardiac function, increased cardiomyocyte death, and greater mortality at 8 weeks post MI [55]. Based on this evidence, we hypothesized that UCP3 deficiency is responsible for impairment of myocardial LCFA oxidation at reperfusion, thereby causing energetic deficiency and impairment of contractile recovery post ischemia.

The goals of the present study were to (1) reexamine in several rodent genetic models the impact of hyperinsulinemia and type 2 diabetes on cardiac UCP3 content, (2) investigate in a newly established, genetically modified rat model the consequences of a partial UCP3 deficiency, mimicking that seen with hyperinsulinemia and insulin resistance, on functional recovery of the heart subjected to I/R; and (3) identify the potential molecular mechanisms at play in the observed phenotype.

Our results demonstrate that myocardial UCP3 deficiency is a common feature of rodents with obesity, insulin resistance and type 2 diabetes, and is linked to the hyperinsulinemic state of the animals. Moreover, our data confirm that partial UCP3 deficiency is sufficient to impair contractile recovery of the rat heart post ischemia, which is linked to mitochondrial dysfunction and decreased capacity to oxidize LCFAs at reperfusion. We also provide evidence that this mitochondrial defect can be bypassed, at least ex vivo, by supplying the heart with a medium-chain fatty acid (MCFA). Taken together, these results support the concept that myocardial UCP3 deficiency is a mechanism for the poor prognosis of type 2 diabetic patients following MI. Our results also expose MCFA supplementation as a potential metabolic intervention to improve recovery of these patients at reperfusion.

## Materials and methods

An expanded “Materials and methods” section is available in the Electronic Supplementary Material.

### Animals

All animals were housed in the Center for Comparative Research animal facilities of the University of Mississippi Medical Center (UMMC). Animals were kept on a 12 h light/12 h dark cycle and fed a standard laboratory rodent diet (Teklad 8640). All procedures were conducted in accordance with the National Institutes of Health’s *Guide for the Care and Use of Laboratory Animals* and approved by the Institutional Animal Care and Use Committee.

Male leptin-deficient *ob/ob* mice (B6.Cg-*Lep*^*ob*^/J), male leptin receptor-deficient *db/db* (B6.BKS(D)-*Lepr*^*db*^/J), and male wild-type controls C57BL/6J mice were all purchased from the Jackson Laboratory. Heart tissue samples from male melanocortin 4 receptor (MC4R)-deficient mice (LoxTB MC4R^−/−^), male mice with leptin receptor deletion in the entire central nervous system (LepR/Nestin-cre), and male MC4R knockout rats (MC4RKO) were obtained from animal colonies housed at UMMC. The UCP3-deficient rats were developed on a Sprague–Dawley background strain (Taconic) by Horizon Discovery using CRISPR/Cas9 technology as described in the Electronic Supplementary Material.

### Blood and plasma parameters

Blood glucose levels were determined with a Contour next blood glucose monitoring system (Bayer Healthcare LLC). Plasma insulin and leptin levels were quantified using the Ultra Sensitive Mouse Insulin (Crystal Chem) and the Quantikine Mouse/Rat Leptin (R&D Systems) ELISA kits.

### Immunoblotting

Details on the immunoblotting procedures and antibodies used can be found in the Electronic Supplementary Material.

### Echocardiography

Cardiac structure and function were assessed by high-frequency echocardiography using a Vevo 3100 imaging system (VisualSonics). Rats were sedated using 2% isoflurane gas and placed on a prewarmed pad with continuous electrocardiographic monitoring. Each echocardiographic session was performed in less than 10 min to minimize the cardiac effects of anesthesia. Two-dimensional B-mode images were obtained from the parasternal long and short axis views. M-Mode images were obtained at the midpapillary muscle level from the short axis view. The Vevo Lab 3.1.1 software cardiac package was used to determine left ventricular (LV) wall dimensions and volume at end-diastole and end-systole, fractional shortening (%), stroke volume, and ejection fraction (%) from an average of five cardiac cycles.

### Working heart preparation

Fifty-one to 61-week-old rats were anesthetized with inhaled isoflurane (3%) and intravenously injected with 200 USP units heparin prior to heart removal. Perfusions were carried out on spontaneously beating hearts following previously established protocols [6]. Detailed descriptions on perfusion conditions and data acquisition are provided in the Electronic Supplementary Material.

### Working heart perfusion protocol

Global myocardial ischemia was induced ex vivo according to the guidelines for experimental models of myocardial ischemia and infarction [44]. Hearts were perfused for 20 min under baseline conditions, after which total, global, normothermic ischemia was induced by clamping both the aortic and the atrial lines on the perfusion system. After 15 min no-flow ischemia, hearts were reperfused for 30 min by unclamping both lines. Measurement of cardiac parameters was reinitiated at 5 min into reperfusion, or at the time point following the recovery of aortic pressure, whichever came first. At the end of the protocol, hearts were freeze-clamped with aluminum tongs cooled in liquid nitrogen and stored at − 80 °C.

### In vivo myocardial infarction and reperfusion

14- to 22-week-old rats were anesthetized by intraperitoneal injection of a mixture of ketamine (100 mg/kg) and xylazine (10 mg/kg). Correct depth of anesthesia was ascertained by lack of toe pinch and palpebral reflexes. Rats were then intubated with a 16G catheter connected to a small animal ventilator (Harvard Apparatus). A small skin cut was made over the left chest. After dissection of the pectoral major and minor muscles, the fourth intercostal space was exposed. A small window was made at the fourth intercostal space to open the pleural membrane and pericardium. Myocardial infarction was induced by ligation of the left anterior descending artery (LAD) using a 4-0 nylon suture tied over a small polyethylene tube. After 45 min ischemia, the tube was removed to allow reperfusion. After 15 min reperfusion, the heart was quickly excised from the chest and placed into ice-cold MSM buffer (220 mM mannitol, 70 mM sucrose, 5 mM MOPS pH 7.4). Following aortic cannulation and retying of the LAD with the suture, the heart was retrogradely perfused with ice-cold MSM to wash blood away. Ice-cold saline supplemented with 0.01% Evans blue was subsequently injected to identify the LV remote and infarct areas. Prior analyses demonstrated that tissue perfusion with 0.01% Evans blue had no effect on mitochondrial function per se.

### Mitochondrial isolation

Heart mitochondria were isolated following previously established protocols with minor modifications [62]. In brief, cardiac tissue was minced with a razor blade in ice-cold MSM buffer supplemented with 1 mg/ml bacterial proteinase type XXIV (Nagarse). The minced tissue was added to 8–10 ml ice-cold isolation buffer (MSM buffer supplemented with 2 mM EDTA and 0.2% fatty acid-free BSA) and homogenized on ice with a glass homogenizer and a Teflon pestle. To minimize damage to the mitochondria, the homogenate was poured off after ~ 5 strokes. After addition of 0.1 mM phenylmethylsulfonyl fluoride (PMSF), the homogenate was centrifuged at 300×*g* for 10 min at 4 ºC. The supernatant was then centrifuged at 3000×*g* for 10 min at 4 ºC, and the mitochondrial pellet washed once in ice-cold isolation buffer. After final resuspension in ice-cold isolation buffer, mitochondrial protein concentration was determined using the DC Protein assay (Bio-Rad). Citrate synthase activity was nine times higher in the mitochondrial fraction when compared to the cytosolic fraction, thereby confirming minimal disruption of mitochondria during the isolation procedure. Methods used for the determination of mitochondrial function and the assessment of respiratory complex activities are provided in the Electronic Supplementary Material.

### Transmission electron microscopy

Left ventricular tissue samples (~ 1 mm^3^) were quickly dissected following euthanasia and immediately fixed in glutaraldehyde. After thin sectioning (70 nm in thickness) and application on copper grids, the stained samples were loaded in a JOEL JEM-1400Plus transmission electron microscope for data acquisition. At least five sections from each sample were examined under transmission electron microscopy. The entire sections were thoroughly viewed at low magnification (300×) for integrity and quality of stained tissues. Mitochondrial ultrastructure was further investigated at high magnifications.

### Isolation and culture of adult rat ventricular myocytes

Adult rat ventricular myocytes (ARVM) were isolated according to a modified version of the method developed by Ackers-Johnson and colleagues [3]. In brief, rats anesthetized with inhaled isoflurane (3%) were intravenously injected with 200 USP units heparin and their hearts subsequently removed and immediately transferred into ice-cold EDTA buffer. Following aortic cannulation, the hearts were retrogradely perfused first with 20 ml of ice-cold EDTA buffer to wash them free of blood, then with 40 ml of ice-cold perfusion buffer, and last with 40 ml of recirculating collagenase buffer pre-warmed at 38 ºC. After proceeding with mechanical dissociation of heart tissue, cell separation by gravity settlement, and calcium re-introduction, ARVM were plated in 24-well culture plates on laminin-coated coverslips. After 24 h in culture, ARVM were subjected to anoxia/reperfusion using the method of enzymatically generated oxygen deficiency [8]. Oxygen depletion was initiated by replacing the culture media with medium containing glucose oxidase (2 U/ml) and catalase (120 U/ml). After 30 min incubation, ARVM were washed three times with fresh culture medium and incubated for another 30 min in culture medium containing 0.5 mM MitoSOX Red (Molecular Probes). At the end of the incubation period, cells were washed three times with PBS and fixed for 10 min in 4% paraformaldehyde solution. The coverslips were then mounted on microscope slides with VECTASHIELD Antifade Mounting Medium with DAPI (Vector Laboratories) and analyzed with an epifluorescence microscope at the manufacturers’ recommended wavelengths.

### Cardiac enzyme activity and malonyl-CoA levels

Activity of the antioxidant enzymes glutathione peroxidase (GPx), catalase (CAT), and superoxide dismutase (SOD) was determined using colorimetric assay kits (Cayman Chemical) following the manufacturer’s protocols. Cardiac tissue malonyl-CoA levels were determined using a rat malonyl coenzyme A ELISA kit (Biomatik). Carnitine palmitoyl transferase (CPT) activity was determined using the isotope forward assay as detailed in the Electronic Supplementary Material.

### Statistical analyses

Data are expressed as mean ± SE. In consideration of sex as a biological variable, UCP3-deficient rats of both sexes were included in the study. In initial experiments involving ex vivo working heart perfusions and isolated mitochondria, all normalized parameters were found to be similar in both sexes and were consequently pooled together for final analyses. Comparisons between two groups were performed using paired or unpaired Student *t* test. Comparisons between multiple experimental groups were made by one-way analysis of variance with Tukey’s post hoc test. A *p* value < 0.05 was considered statistically significant. All the analyses were performed with GraphPad Prism version 7.

## Results

### Cardiac UCP3 content is decreased in monogenic mouse and rat models of obesity, insulin resistance and type 2 diabetes

**Fig. 1:**
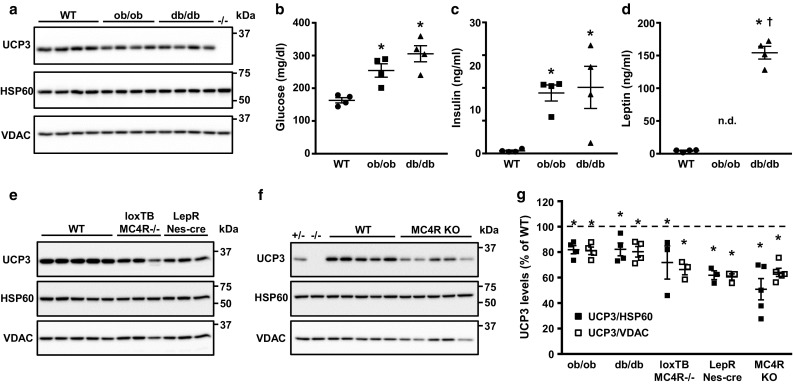
Cardiac UCP3 levels are decreased in rodent models of obesity, insulin resistance and type 2 diabetes. **a** Immunoblotting revealed a decrease in UCP3 protein levels in hearts of male *ob*/*ob* mice (*n* = 4) and male *db*/*db* mice (*n* = 4) when compared to wild-type (WT) male C57BL/6J control mice (*n* = 4). Both *ob*/*ob* and *db*/*db* mice were hyperglycemic (**b**) and hyperinsulinemic (**c**). **d** *db*/*db* mice displayed severe hyperleptinemia, while plasma leptin levels were undetectable in *ob*/*ob* mice. **e** UCP3 protein levels were also decreased in hearts from type 2 diabetic male LoxTB MC4R^−/−^ mice (*n* = 3) and male LepR/Nestin-cre mice (*n* = 3). **f** Cardiac UCP3 protein levels were similarly decreased in hearts from obese and insulin-resistant MC4RKO rats (*n* = 5). **g** The decrease in cardiac UCP3 protein levels in these animals ranged between 20 and 49%. Similar results were obtained when normalizing UCP3 levels to the ubiquitous cellular protein HSP60 or to the mitochondrial protein VDAC. Data are presented as mean ± SE. *p < 0.05 vs. WT animals; ^†^*p* < 0.05 vs. *ob*/*ob* mice

Because of the conflicting reports on the effect of non-insulin-dependent diabetes on UCP3 expression in the heart, we first set out to quantify cardiac UCP3 levels in a panel of rodent models of obesity, insulin resistance and type 2 diabetes. We used a recently developed monoclonal antibody that recognizes both UCP1 and UCP3. Specificity for UCP3 was validated on tissue protein lysates from UCP3 knockout rats (Supplemental Fig. 1c). Cardiac UCP3 content was decreased by 20% in two of the most widely used models of obesity and type 2 diabetes, the *ob/ob* and *db/db* mice (Fig. 1a, g). As expected, although both *ob/ob* and *db/db* mice displayed severe hyperglycemia and hyperinsulinemia (Fig. 1b, c), only *db/db* mice developed hyperleptinemia, while circulating leptin levels were below detection range for *ob/ob* mice (Fig. 1d). An even stronger decrease in cardiac UCP3 content was observed in the LoxTB MC4R^−/−^ mice (28–34% decrease) and LepR/Nestin-cre mice (38–40% decrease; Fig. 1e, g), two other models of type 2 diabetes which are also characterized by severe obesity, insulin resistance, hyperinsulinemia, and glucose intolerance [7, 49]. A strong 36–49% decrease in cardiac UCP3 content was also observed in the MC4RKO rat (Fig. 1f, g), a rodent model with marked obesity, insulin resistance, and hyperinsulinemia [65]. Thus, cardiac UCP3 content is consistently decreased in obese, insulin-resistant, and type 2 diabetic animals, and a common denominator to this decrease is the development of hyperinsulinemia mediated by insulin resistance.

### Partial loss of UCP3 impairs LCFA oxidation and contractile recovery in rat hearts post ischemia

**Fig. 2:**
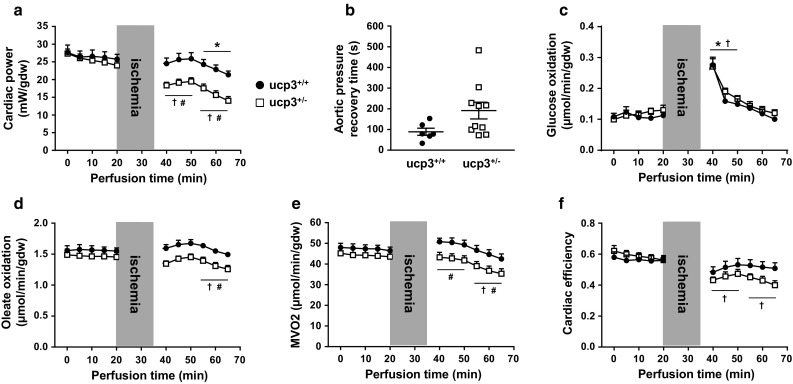
Partial loss of UCP3 impairs metabolic and functional recovery of rat hearts at reperfusion. Isolated hearts from ucp3^+/+^ control rats (black circles, *n* = 6–8) and ucp3^+/−^ rats (open squares, *n* = 10–11) were perfused in the working mode in the presence of 5.5 mM glucose and 0.8 mM of the LCFA oleate as substrates. Cardiac power (**a**), time to recovery of aortic pressure post ischemia (**b**), rates of glucose oxidation (**c**), rates of oleate oxidation (**d**), myocardial oxygen consumption (**e**), and cardiac efficiency (**f**) were determined as described in the “Methods” section. Data are presented as mean ± SE. *p < 0.05 ucp3^+/+^ vs. baseline; ^†^*p *< 0.05 ucp3^+/−^ vs. baseline. ^#^*p* < 0.05 vs. ucp3^+/+^

To recapitulate the decrease in UCP3 levels caused by hyperinsulinemia, insulin resistance and type 2 diabetes, CRISPR-Cas9 genome editing was used to inactivate one copy of the UCP3 gene in Sprague–Dawley rats (ucp3^+/−^). Mutant male and female adult rats had normal cardiac functional and structural parameters when compared to their wild-type ucp3^+/+^ counterparts (Supplemental Table 1). Isolated working hearts from both groups of animals also displayed similar functional and metabolic parameters when perfused under baseline conditions with oleate as the exogenous source of fatty acids for β-oxidation (Fig. 2; Table 1). But while cardiac power from ucp3^+/+^ hearts recovered to near-baseline values following 15 min of total global normothermic ischemia, hearts from ucp3^+/−^ animals experienced a rapid and sustained impairment in contractile function at reperfusion (Fig. 2a). Time to recovery of aortic pressure post ischemia also tended to increase for the ucp3^+/−^ hearts (*p* = 0.08; Fig. 2b). Myocardial rates of glucose oxidation increased transiently at early reperfusion for both groups and then declined to pre-ischemia rates during late reperfusion (Fig. 2c). However, rates of long-chain fatty acid oxidation decreased significantly in ucp3^+/−^ hearts during the late reperfusion period, as compared to the pre-ischemia rates (Fig. 2d). As an apparent consequence, both M*V*_*O*2_ and cardiac efficiency dropped significantly for ucp3^+/−^ hearts during reperfusion (Fig. 2e, f).

**Table 1:** Functional and metabolic parameters of isolated working hearts

Ucp3		Baseline (0–20 min)			Early reperfusion (35–50 min)			Late reperfusion (50–65 min)		
		OL	OA	OL/OA	OL	OA	OL/OA	OL	OA	OL/OA
+/+	CP	26.5 ± 0.7	25.3 ± 0.6	26.6 ± 0.9	25.3 ± 0.9	22.8 ± 1.1	23.0 ± 1.3	22.8 ± 0.7*	24.6 ± 1.1	22.6 ± 1.2
	GO	111 ± 6	80 ± 7^**†**^	85 ± 5^**†**^	195 ± 18*	115 ± 10^**†**^	148 ± 13*	118 ± 7	91 ± 8^**†**^	102 ± 10
	FAO^a^	1563 ± 29	4853 ± 190	348 ± 22^**†**^	1641 ± 36	4562 ± 212	502 ± 43*^,**†**^	1561 ± 23	4762 ± 149	514 ± 33*^,**†**^
	M*V*_*O2*_	47.3 ± 0.8	44.8 ± 0.8	42.8 ± 1.7^**†**^	50.1 ± 1.1	45.2 ± 0.9	46.8 ± 2.3	44.6 ± 1.3	41.1 ± 1.3	43.2 ± 2.0
	CE	0.56 ± 0.01	0.57 ± 0.02^**#**^	0.63 ± 0.02^**†**^	0.51 ± 0.02	0.51 ± 0.02	0.50 ± 0.02*	0.52 ± 0.02	0.60 ± 0.03^**†**^	0.53 ± 0.03*
+/−	CP	25.5 ± 0.5	24.1 ± 0.6	25.0 ± 0.7	19.0 ± 0.6*	21.3 ± 0.8^**#**^	23.9 ± 0.7^**†**^	15.7 ± 0.7*	22.2 ± 0.7^**†**^	21.7 ± 0.8^**†**^
	GO	117 ± 6	78 ± 4^**†**,**#**^	113 ± 7	207 ± 12*	132 ± 8*^,**†**^	160 ± 9*^,**†**^	131 ± 7	88 ± 6^**†**^	106 ± 7^**†**^
	FAO^a^	1470 ± 36	5080 ± 80	397 ± 9^**†**^	1410 ± 28	4818 ± 126	554 ± 24*^,**†**^	1320 ± 34*	4888 ± 107	540 ± 20*^,**†**^
	M*V*_*O2*_	44.2 ± 1.2	43.6 ± 1.0^**#**^	48.5 ± 0.6^**†**^	42.5 ± 1.3	45.0 ± 1.4^**#**^	49.9 ± 0.7^**†**^	37.0 ± 1.3*	41.2 ± 1.1^**†**,**#**^	45.2 ± 0.7^**†**^
	CE	0.59 ± 0.01	0.56 ± 0.01	0.51 ± 0.02^**†**^	0.45 ± 0.02*	0.48 ± 0.02*	0.48 ± 0.02	0.43 ± 0.02*	0.55 ± 0.02^**†**,**#**^	0.48 ± 0.02

Hearts were subjected to I/R and perfused with the LCFA oleate (OL), the MCFA octanoate (OA), or an equimolar mixture of both fatty acids (OL/OA) as described in the “Materials and methods” section. Data are presented as mean ± SE

*CE* cardiac efficiency, *CP* cardiac power (mW/gdw), *GO* glucose oxidation (nmol/min/gdw), *FAO* fatty acid oxidation (nmol/min/gdw), *MV*_*O2*_ myocardial oxygen consumption (µmol/min/gdw)

*p < 0.05 vs. baseline; ^†^*p* < 0.05 vs. OL; ^#^*p* < 0.05 vs. OL/OA

^a^Only rates of oleate oxidation were measured in the OL/OA condition

### Partial loss of UCP3 impairs mitochondrial LCFA oxidation in rat hearts post ischemia

**Fig. 3:**
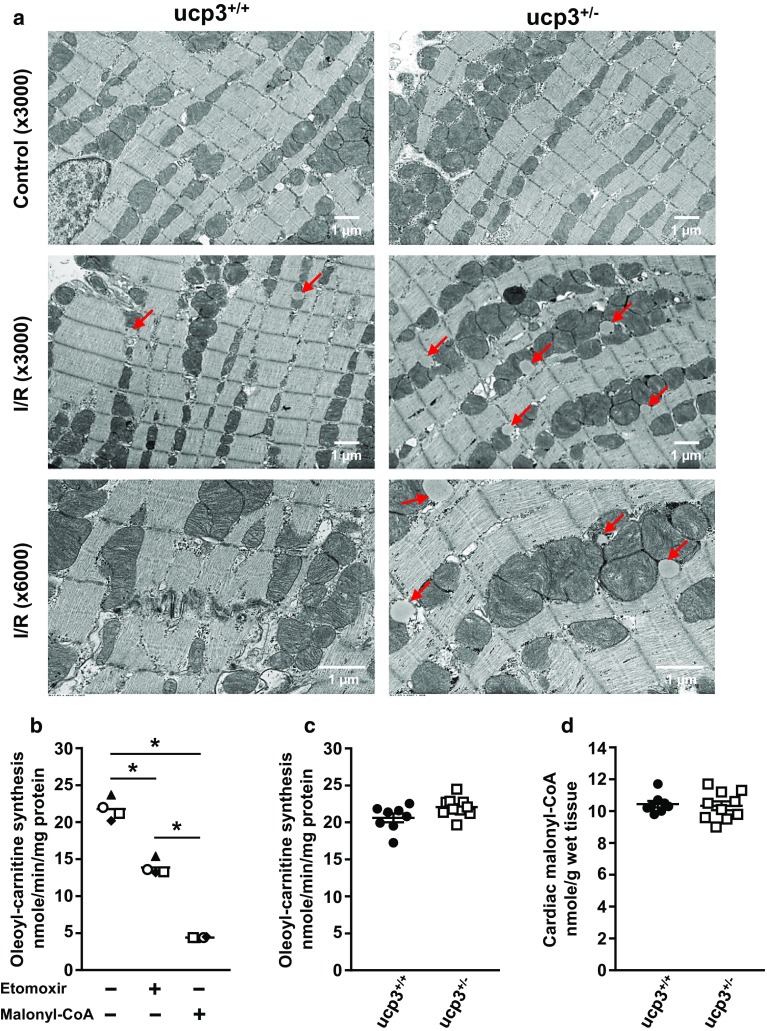
UCP3 deficiency induces cytosolic lipid accumulation in cardiomyocytes following I/R. **a** Electron micrographs of cardiac tissue from rat hearts subjected to in vivo MI/reperfusion. The ultrastructure of cardiomyocytes from the remote (control) and the infarct (I/R) areas is presented. Red arrows indicate lipid droplets. Representative photomicrographs of *n *= 3 rats per group and per condition are shown. **b** The inhibition of myocardial CPT1 activity by incubation of heart tissue lysates (*n* = 4) with the known CPT1 inhibitors etomoxir (100 µM) and malonyl-CoA (200 µM) confirmed the specificity of the assay. CPT1 activity (**c**) and malonyl-CoA levels (**d**) were determined in hearts from ucp3^+/+^ control rats (black circles, *n *= 8) and ucp3^+/−^ rats (open squares, *n *= 10) following I/R. Data are presented as mean ± SE. *p < 0.05

In order to determine whether an impairment of LCFA oxidation in ucp3^+/−^ hearts may also occur in vivo in response to I/R, male and female rats were subjected to 45 min of acute MI followed by 15 min of reperfusion. Electron microscopy visualization of LV cardiac tissue from the remote area did not reveal any difference in cardiomyocyte ultrastructure among genotypes. However, in the infarct area, the number and size of cytosolic lipid droplets increased noticeably in cardiomyocytes from ucp3^+/−^ animals (Fig. 3a).

Carnitine palmitoyltransferase I (CPT1), the enzyme controlling the transport of LCFAs in the mitochondrial matrix, is regarded as the rate-limiting enzyme in fatty acid β-oxidation [26]. The activity of CPT1 in the heart is highly regulated and can be strongly inhibited by compounds and metabolites such as malonyl-CoA (Fig. 3b). However, the activity of CPT1 and malonyl-CoA content of ucp3^+/−^ hearts subjected to I/R were similar to that of ucp3^+/+^ hearts (Fig. 3c, d), suggesting that the impairment of LCFA oxidation occurs downstream of their mitochondrial import.

**Fig. 4:**
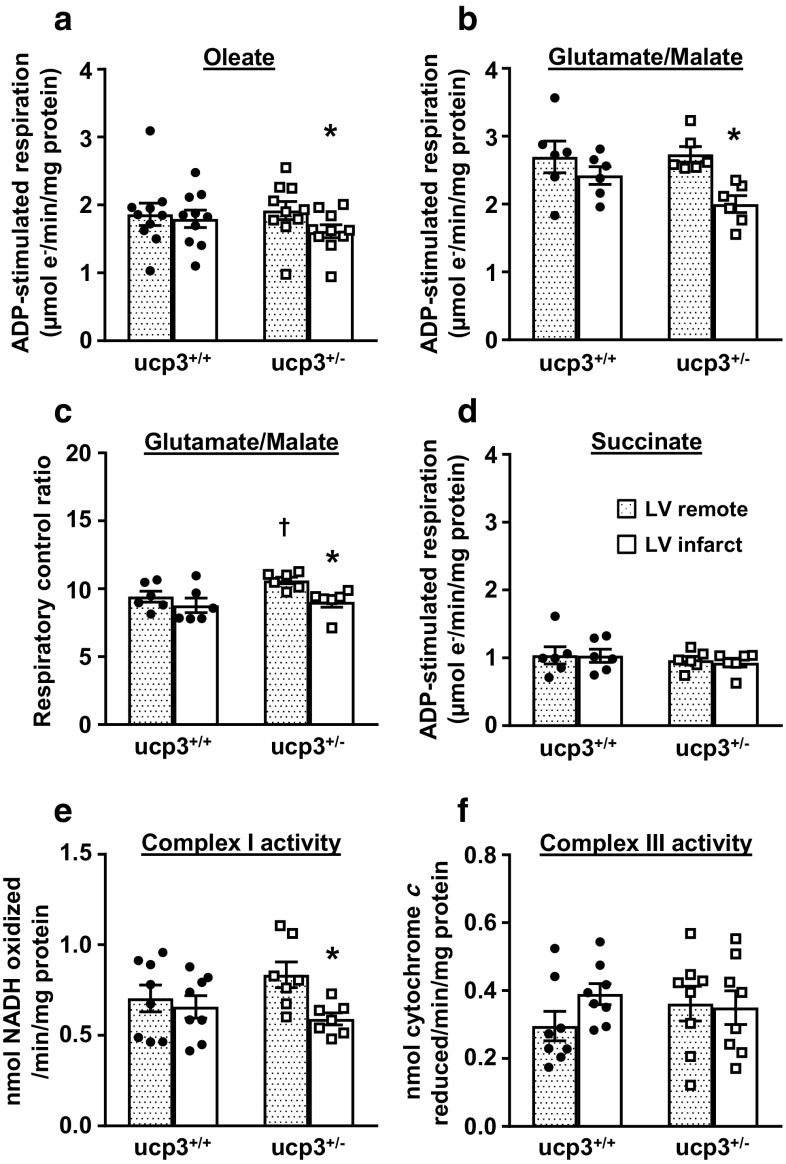
Partial loss of UCP3 impairs mitochondrial function post ischemia. Hearts from ucp3^+/+^ control rats (black circles, *n* = 6–10) and ucp3^+/−^ rats (open squares, *n* = 6–10) were subjected to MI/reperfusion. Mitochondrial function from the remote region (dotted bars) and the infarct area (open bars) were then analyzed in parallel to determine the rates of ADP-stimulated respiration in the presence of 0.5 mM oleate (**a**), the rates of ADP-stimulated respiration in the presence of 20 mM glutamate and 10 mM malate (**b**), the respiratory control ratio in the presence of 20 mM glutamate and 10 mM malate (**c**), and the rates of ADP-stimulated respiration in the presence of 20 mM succinate (**d**). The activity of the respiratory complex I (**e**) and complex III (**f**) was determined using broken mitochondria prepared as described in the “Methods” section. Data are presented as mean ± SE. *p < 0.05 vs. LV remote. ^†^*p* < 0.05 vs. ucp3^+/+^

To further investigate whether the impairment of LCFA oxidation in ucp3^+/−^ hearts is linked to a mitochondrial defect occurring at reperfusion, mitochondria were isolated from LV cardiac tissue after MI/reperfusion and their respiratory capacity in presence of various substrates compared. In mitochondria isolated from the remote area of both groups, the rate of ADP-stimulated respiration of oleate was similar (Fig. 4a). In mitochondria isolated from the infarct area of ucp3^+/+^ hearts, the rate of ADP-stimulated respiration of oleate was the same as remote levels, but the rate decreased by 16% in the ucp3^+/−^ group (Fig. 4a). A similar decrease was observed for ADP-stimulated respiration of glutamate/malate by ucp3^+/−^ mitochondria isolated from the infarct area (Fig. 4b, c). However, such a decrease was not observed when using the complex II substrate succinate, although ADP-stimulated respiration was overall slower in this condition (Fig. 4d). Consistent with the respiratory substrate analyses, complex I activity, but not complex III activity, was decreased in ucp3^+/−^ mitochondria subjected to I/R (Fig. 4e, f). Complex IV activity (data not shown) remained unaltered for both genotypes before and after ischemia.

### Mitochondrial ROS production increases in UCP3-deficient hearts post ischemia

**Fig. 5:**
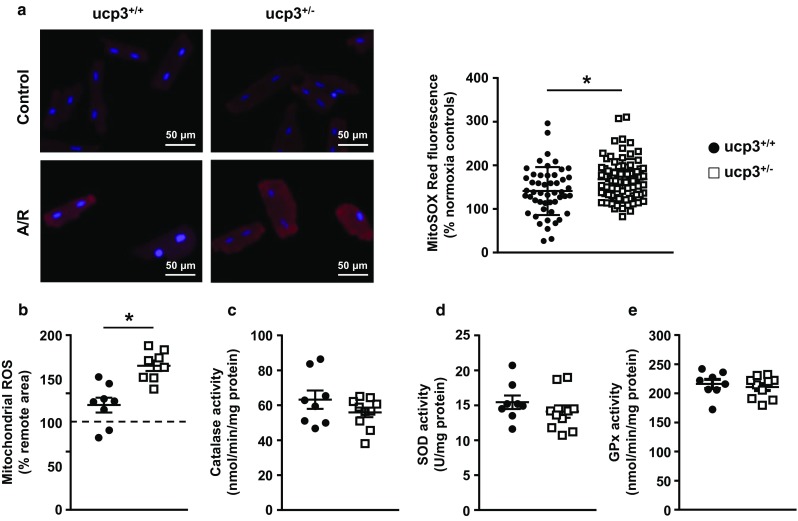
Partial loss of UCP3 increases mitochondrial ROS production post ischemia. **a** Cultured adult rat ventricular myocytes isolated from ucp3^+/+^ (black circles, *n* = 50) and ucp3^+/−^ (open squares, *n* = 79) rats were exposed to 30 min anoxia followed by 30 min reoxygenation (A/R) in the presence of the superoxide probe MitoSOX Red. **b** Cardiac mitochondria were isolated from the remote regions and the infarct areas of ucp3^+/+^ (black circles, *n* = 8) and ucp3^+/−^ (open squares, *n* = 8) rats subjected to MI/reperfusion and incubated with 0.5 mM oleate in the presence of the hydrogen peroxide probe Amplex Red. **c**–**e** The activity of the antioxidant enzymes catalase, superoxide dismutase (SOD), and glutathione peroxidase (Gpx) was measured in hearts from ucp3^+/+^ (*n* = 8) and ucp3^+/−^ (*n* = 10) rats. Data are presented as mean ± SE. *p < 0.05

Mitochondrial reactive oxygen species (ROS) production has previously been reported to increase in hearts of ucp3^−/−^ mice and to mediate cardiac injury following MI [53, 55]. In order to determine whether ROS generation is similarly exacerbated by partial UCP3 deficiency in rat cardiomyocytes in response to acute hypoxic stress, ARVM were subjected to 30 min anoxia followed by 30 min reoxygenation in the presence of the mitochondrial superoxide probe MitoSOX. Compared to cells maintained under normoxic conditions, superoxide generation increased on average by 41% in ucp3^+/+^ cardiomyocytes subjected to anoxia-reoxygenation. Cells with decreased amounts of UCP3 amplified ROS generation at reperfusion, with superoxide levels increasing by 67% on average (Fig. 5a). An increase in ROS generation was also observed in UCP3-deficient mitochondria isolated from LV cardiac tissue subjected to I/R when using oleate as the respiratory substrate (Fig. 5b). This increase in mitochondrial ROS generation was not accompanied by a compensatory increase of cellular antioxidant defenses in UCP3-deficient hearts as the activity of CAT, SOD, and GPx remained unchanged when compared to cardiac tissue from ucp3^+/+^ rats (Fig. 5c–e).

### Hearts from UCP3-deficient rats supplied with octanoate are protected from mitochondrial and contractile dysfunction at reperfusion

**Fig. 6:**
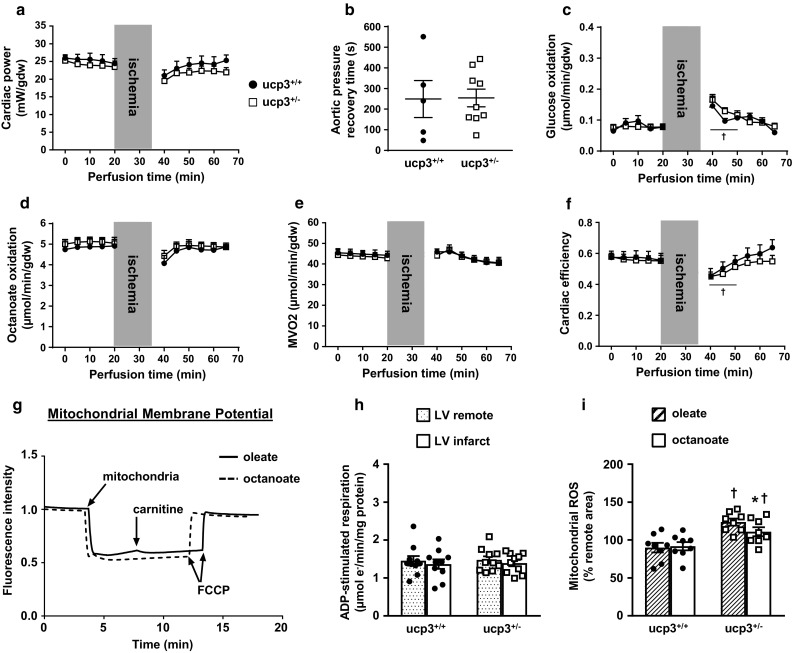
Octanoate promotes contractile recovery and preserves mitochondrial function in ucp3^+/−^ hearts at reperfusion. Isolated hearts from ucp3^+/+^ control rats (black circles, *n* = 5) and ucp3^+/−^ rats (open squares, *n* = 9–10) were perfused in the working mode in the presence of 5.5 mM glucose and 0.8 mM of the MCFA octanoate as substrates. Cardiac power (**a**), time to recovery of aortic pressure post ischemia (**b**), rates of glucose oxidation (**c**), rates of octanoate oxidation (**d**), myocardial oxygen consumption (**e**), and cardiac efficiency (**f**) were determined as described in the “Methods” section. Data are presented as mean ± SE. ^†^*p* < 0.05 ucp3^+/−^ vs. baseline. **g** Mitochondrial membrane potentials measured with the fluorescent probe Safranin O when using 0.5 mM oleate (plain line) or 0.5 mM octanoate (hatched line) as substrates for ADP-stimulated respiration. FCCP, Carbonyl cyanide 4-(trifluoromethoxy)phenylhydrazone. **h** ADP-stimulated respiration in the presence of 0.5 mM octanoate was measured for mitochondria isolated from the remote region (dotted bars) and the infarct area (open bars) of ucp3^+/+^ (*n* = 10) and ucp3^+/−^ (*n* = 10) rat hearts subjected to MI/reperfusion. **i** Comparison of ROS generation in mitochondria incubated with 0.5 mM oleate (hatched bars) or 0.5 mM octanoate (open bars) following isolation from the infarct area of ucp3^+/+^ (*n* = 8) and ucp3^+/−^ (*n* = 8) rat hearts subjected to MI/reperfusion. Data are presented as mean ± SE. *p < 0.05 vs. oleate; ^†^*p* < 0.05 vs. ucp3^+/+^

The I/R protocol was repeated on isolated working hearts from both genotypes, this time replacing oleate by the MCFA octanoate as the exogenous source of fatty acids for β-oxidation (Fig. 6a–f). Under baseline conditions, ucp3^+/+^ hearts perfused with octanoate had similar cardiac power as when perfused with oleate (Table 1). Following ischemia, the contractile function of ucp3^+/+^ hearts perfused with octanoate recovered completely (Fig. 6a) although the time to recovery of aortic pressure (Fig. 6b) tended to increase in comparison to hearts perfused with oleate (249 ± 90 vs. 89 ± 17 s; *p *= 0.08). Octanoate was readily oxidized by the ucp3^+/+^ hearts before and after ischemia (Fig. 6d). In comparison to oleate, perfusion with octanoate led to a 28% decrease in myocardial rates of glucose oxidation at baseline, as well as 41% and 23% decreases during the early and late reperfusion periods, respectively (Table 1).

All cardiac parameters for ucp3^+/−^ hearts perfused with octanoate evolved comparably to those of ucp3^+/+^ hearts perfused with octanoate (Fig. 6a–f). Cardiac power, M*V*_*O*2_, and cardiac efficiency of ucp3^+/−^ hearts perfused with octanoate were all improved in late reperfusion period when compared to ucp3^+/−^ hearts reperfused with oleate (Table 1). This improvement occurred in spite of a time to recovery of aortic pressure similar to the one observed on perfusion with oleate (254 ± 43 vs. 192 ± 41 s; *p* = 0.31).

In mitochondria isolated from ucp3^+/+^ hearts, the MCFA octanoate tended to decrease ADP-stimulated respiration rates when compared to the LCFA oleate (1.45 ± 0.12 vs. 1.86 ± 0.16 µmol e^−^/min/mg protein; *p* = 0.06). This had, however, no detrimental effect on mitochondrial energization since the mitochondrial membrane potential was comparable to the one obtained with oleate (Fig. 6g). In further contrast to oleate, the ADP-stimulated respiration rate of ucp3^+/−^ cardiac mitochondria oxidizing octanoate was preserved following MI-reperfusion (Fig. 6h). Although mitochondrial ROS generation during octanoate oxidation was still amplified by UCP3 deficiency at reperfusion, the increase in ROS was 13% lower than that observed in the comparable experiment using oleate as the respiratory substrate (Fig. 6i).

### Octanoate supplementation preserves mitochondrial oleate oxidation and contractile recovery of UCP3-deficient rat hearts at reperfusion

**Fig. 7:**
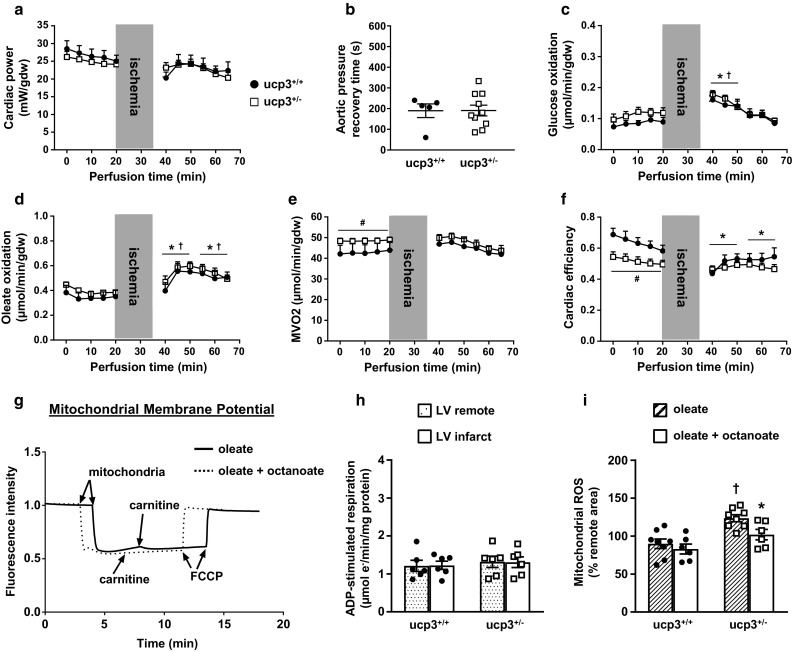
Octanoate supplementation promotes contractile recovery and preserves oleate oxidation and mitochondrial function in ucp3^+/−^ hearts at reperfusion. Isolated hearts from ucp3^+/+^ control rats (black circles, *n* = 5) and ucp3^+/−^ rats (open squares, *n* = 10–13) were perfused in the working mode in the presence of 5.5 mM glucose, 0.4 mM oleate, and 0.4 mM octanoate as substrates. Cardiac power (**a**), time to recovery of aortic pressure post ischemia (**b**), rates of glucose oxidation (**c**), rates of oleate oxidation (**d**), myocardial oxygen consumption (**e**), and cardiac efficiency (**f**) were determined as described in the “Methods” section. Data are presented as mean ± SE. *p < 0.05 ucp3^+/+^ vs. baseline; ^†^*p *< 0.05 ucp3^+/−^ vs. baseline; ^#^*p* < 0.05 vs. ucp3^+/+^. **g** Mitochondrial membrane potentials measured with the fluorescent probe Safranin O when using 0.5 mM oleate (plain line) or 0.5 mM of an equimolar mixture of oleate and octanoate (dotted line) as substrates for ADP-stimulated respiration. FCCP, Carbonyl cyanide 4-(trifluoromethoxy)phenylhydrazone. **h** ADP-stimulated respiration in the presence of 0.5 mM of an equimolar mixture of oleate and octanoate was measured for mitochondria isolated from the remote region (dotted bars) and the infarct area (open bars) of ucp3^+/+^ (*n* = 6) and ucp3^+/−^ (*n* = 6) rat hearts subjected to MI/reperfusion. **i** Comparison of ROS generation in mitochondria incubated with 0.5 mM oleate (hatched bars) or 0.5 mM of an equimolar mixture of oleate and octanoate (open bars) following isolation from the infarct area of ucp3^+/+^ (*n* = 6–8) and ucp3^+/−^ (*n* = 6–8) rat hearts subjected to MI/reperfusion. Data are presented as mean ± SE. *p < 0.05 vs. oleate; ^†^*p* < 0.05 vs. ucp3^+/+^

Similar to the use of octanoate alone, the reperfusion of ucp3^+/+^ hearts with a mixture of 50% octanoate and 50% oleate maintained cardiac power under basal conditions (Table 1). Utilization of the octanoate/oleate mixture decreased myocardial rates of glucose oxidation by 26% and also led to a 4.5-fold decrease in myocardial rates of oleate oxidation at baseline. These changes in substrate oxidation were accompanied by a decrease in M*V*_*O*2_ and an improvement in cardiac efficiency for the ucp3^+/+^ hearts at baseline (Table 1). Following ischemia, contractile recovery of ucp3^+/+^ hearts was complete and sustained over the whole reperfusion period, although the recovery of aortic pressure was still delayed when compared to oleate (190 ± 33 vs. 89 ± 17 s; *p* = 0.01). Interestingly, the recovery of cardiac power occurred in the presence of an increase in rates of oleate oxidation (Fig. 7d). There was also a reduction in cardiac efficiency (Fig. 7f). For the UCP3-deficient hearts, the recovery of all cardiac parameters at reperfusion followed that of the ucp3^+/+^ hearts (Fig. 7a–f). Consequently, and as for perfusion with octanoate alone, the recovery of cardiac power and M*V*_*O*2_ was improved for ucp3^+/−^ hearts when compared to perfusion with oleate alone. Again, complete recovery of cardiac parameters was independent from improvement of time to recovery of aortic pressure post-ischemia (191 ± 26 s; *p* = 0.99 vs. oleate).

The incubation of mitochondria isolated from ucp3^+/+^ hearts with the octanoate/oleate mixture resulted in a 35% decrease in the rate of ADP-stimulated respiration when compared to incubation with oleate alone (1.21 ± 0.14 vs. 1.86 ± 0.16 µmol e^−^/min/mg protein; *p* = 0.01). However, and as for mitochondria incubated with octanoate alone, the decrease in the rate of ADP-stimulated respiration did not lead to a decreased mitochondrial membrane potential (Fig. 7g). The combined supply of MCFA and LCFA also prevented a drop in the rate of ADP-stimulated respiration for ucp3^+/−^ cardiac mitochondria subjected to MI-reperfusion (Fig. 7h). Moreover, the burst of ROS generated post-ischemia by ucp3^+/−^ mitochondria was attenuated when compared to ucp3^+/−^ mitochondria supplied with oleate alone, so that ROS levels were now comparable to that produced by ucp3^+/+^ mitochondria (Fig. 7i).

## Discussion

The goals of this study were to reexamine myocardial UCP3 expression in several rodent models of obesity, insulin resistance and type 2 diabetes, and to determine the consequences for cardiac adaptation to I/R. We found that UCP3 protein levels were systematically decreased in the hearts of mouse and rat models of obesity, insulin resistance and type 2 diabetes. Through targeted mutation of one UCP3 gene copy in rats, we showed that partial myocardial UCP3 deficiency, resembling that which occurs with type 2 diabetes, was sufficient to severely impair the contractile recovery of ex vivo perfused hearts subjected to I/R. The decrease in contractile recovery was linked to mitochondrial dysfunction characterized by increased ROS generation, decreased respiratory complex I activity, and impaired LCFA oxidation capacity. We also demonstrated that supplying large amounts of the MCFA octanoate, alone or combined with the LCFA oleate, slowed the flow of electrons through the respiratory chain and decreased the generation of ROS in ucp3^+/−^ cardiac mitochondria. The use of octanoate also led to a decrease in myocardial rates of glucose oxidation and improved LCFA oxidation capacity at reperfusion. At the functional level, MCFA supplementation promoted complete recovery of contractile function for ucp3^+/−^ hearts following ischemia.

### Impact of obesity, insulin resistance and type 2 diabetes on cardiac UCP3 levels

The transcription factor peroxisome proliferator-activated receptor (PPAR)-α potently activates myocardial UCP3 expression in response to increased delivery of LCFAs to cardiomyocytes [45]. Because obesity and diabetes are associated with an increased supply of circulating fatty acids to the heart, myocardial UCP3 levels should increase under those conditions [51]. In fact, there is considerable evidence for increased myocardial UCP3 levels with high-fat feeding and with type 1 diabetes [17, 23, 28]. However, the impact of type 2 diabetes on myocardial UCP3 expression has remained controversial [10, 11]. Insulin resistance and hyperinsulinemia play a central role in the metabolic disturbances associated with type 2 diabetes [61]. We previously reported a decrease in cardiac UCP3 protein levels in experimental rat and mouse models of hyperinsulinemia and insulin resistance [31, 32]. We also demonstrated that hyperinsulinemia triggers insulin resistance in the heart, which in turn induces lipogenic transcription factor SREBP-1 and subsequent down-regulation of UCP3 expression [31]. In the present study, we extended our investigations to monogenic rodent models of obesity, insulin resistance and type 2 diabetes and found that cardiac UCP3 protein levels were reduced between 20% and 49%. Our findings of decreased UCP3 protein expression in the hearts of *ob*/*ob* mice are consistent with a report from the Abel group [11]. Our results are also in line with the respective 46% and 35% decreases in UCP3 protein content observed in skeletal muscle of prediabetic and type 2 diabetic humans [59]. Thus, the data accumulated so far suggest that type 2 diabetes inhibits UCP3 expression in myocytes, thereby leading to partial UCP3 protein deficiency in the heart.

### Effect of partial UCP3 deficiency on cardiac recovery following I/R

Rats fed a high-sucrose diet become insulin resistant and display, on average, a 40% decrease in cardiac UCP3 content. Isolated working hearts from these rats exhibit a significant decrease in rates of LCFA oxidation and a severe impairment of contractile recovery when subjected to I/R [33]. The Russell group and the Esposito group demonstrated a cardioprotective role for UCP3 for ischemic heart disease. Using ucp3^−/−^ mice, these groups found a decrease in myocardial ATP content in response to I/R, a 50% increase in infarct size post MI, higher rates of apoptosis, and worsening of contractile function when compared to wild-type mice. Both teams posited that increased ROS were responsible for the increased failure of hearts lacking UCP3 [53, 55]. However, insulin resistance and type 2 diabetes are associated with only a partial deficiency in UCP3, and it remained unknown if a partial deficiency was sufficient to make the heart more vulnerable to MI and I/R injury. Here, we have used CRISPR/Cas9-targeted mutagenesis to inactivate one copy of the UCP3 gene in rats to explore this question. Although hearts from ucp3^+/−^ rats had normal metabolic and contractile functions under basal perfusion conditions, myocardial rates of LCFA oxidation and cardiac power were dramatically impaired in response to I/R. An increased accumulation of lipid droplets in ucp3^+/−^ cardiomyocytes indicated impairment of LCFA utilization at reperfusion. Thus, partial UCP3 deficiency is sufficient to impair myocardial LCFA metabolism and contractile recovery at reperfusion.

### Partial loss of UCP3 and impairment of myocardial LCFA oxidation

LCFAs are the preferred substrate for energy production in the heart when the supply of oxygen is not limited [64]. There exists compelling evidence that the mild uncoupling activity of UCP3 is a consequence of its regulation of fatty acid utilization [14]. Thus, mild overexpression of UCP3 increases LCFA oxidation in rat L6 myocytes and in skeletal muscle of mice [9, 46]. The accumulation of lipid droplets in ucp3^+/−^ cardiomyocytes suggested either an impairment of the import of LCFA into mitochondria or decreased metabolic flux further downstream, in β-oxidation or oxidative phosphorylation. The fact that CPT1 activity remained unchanged in perfused hearts pointed towards the latter.

**Fig. 8:**
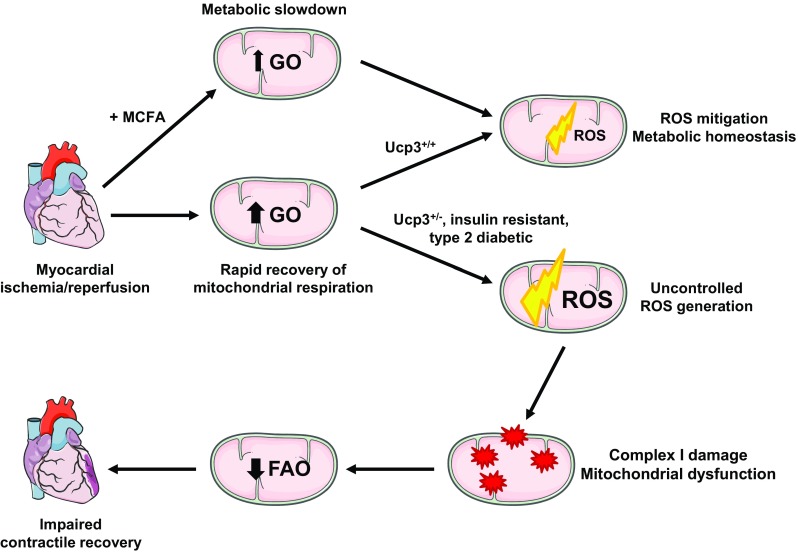
Proposed mechanism for the postischemic impairment of cardiac contractile recovery caused by UCP3 deficiency and its prevention with medium-chain fatty acid (MCFA) supplementation. FAO fatty acid oxidation, GO glucose oxidation

During I/R, loss of pH gradient and membrane potential both contribute to enhanced superoxide generation by cardiac mitochondria [38]. As mentioned above, ROS production is increased in cardiac mitochondria from mice lacking UCP3 in response to I/R [53, 67]. Consistent with those reports, we observed increased mitochondrial ROS generation in UCP3-deficient rat cardiomyocytes subjected to a brief period of oxygen deprivation. Because UCP3 deficiency was not associated with a compensatory increase in cellular antioxidant defenses, the increased burst of ROS is likely to have caused oxidative damage within mitochondria during reperfusion. Subsarcolemmal mitochondria, which normally contain higher UCP3 levels, are especially affected by I/R-induced damage due to their exposure to higher oxygen levels [27, 37]. In particular, complex I has been identified as a primary target for oxidative damage in the mammalian heart post ischemia, resulting in decreased activity or outright inactivation of some of the enzyme [30, 54, 56]. Indeed, we found that complex I activity was decreased in ucp3^+/−^ mitochondria following I/R. Inhibition of complex I leads to NADH accumulation and to an increase in cellular NADH/NAD^+^ [58]. The NADH/NAD^+^ balance exerts tight control over β-oxidation flux so that fatty acid oxidation is inhibited when NADH/NAD^+^ rises [26]. Hence, our findings suggest that the increased burst of mitochondrial ROS during reperfusion of the ucp3^+/−^ hearts may have inactivated some of the complex I, increased NADH/NAD^+^, and, thereby, decreased the flux of LCFA through β-oxidation. Decreased rates of oxidative phosphorylation driven by LCFA would, in turn, limit ATP production and the contractile recovery of the ucp3^+/−^ heart (Fig. 8).

### MCFA supplementation as a therapeutic intervention for the UCP3-deficient heart post ischemia

We have shown that the contractile dysfunction induced ex vivo by I/R in hearts of insulin-resistant rats could be prevented by replacing the LCFA oleate with the MCFA octanoate in the perfusion buffer [33]. In the present study, we also observed a protective effect of octanoate for the ucp3^+/−^ heart. Moreover, perfusing hearts with an equimolar mixture of octanoate and oleate also promoted full recovery of contractile function. Although LCFAs constitute the bulk of circulating fatty acids and the primary substrate for the healthy mammalian heart, MCFAs can be readily oxidized by the heart as well. Our findings are consistent with previous studies demonstrating that MCFAs can restore function in hearts that have an impaired capacity to utilize LCFAs. The metabolism of MCFAs in stressed hearts has been shown to restore a normal glucose-to-fat oxidation balance and to increase ATP generation [41]. In hearts from spontaneously hypertensive rats subjected to an acute increase in workload, MCFA oxidation compensates for decreased capacity to utilize exogenous LCFAs [42]. In hypertrophied hearts from aortic-constricted rats, addition of octanoate (C8:0) to palmitate (C16:0) in the perfusate leads to increased ATP generation [5]. Moreover, MCFAs greatly enhance post-ischemic recovery of hearts from CD36-null mice, which have decreased capacity to take up LCFAs [36].

Four important features distinguished hearts that were perfused in the presence of octanoate from hearts that were perfused with oleate alone: (1) decreased rates of glucose oxidation, especially in the first minutes of reperfusion; (2) slower mitochondrial rates of ADP-stimulated respiration; (3) increased time to recovery of aortic pressure post-ischemia, and (4) mitigation of mitochondrial ROS generation during reperfusion in UCP3-deficient hearts. Taken together, those features seem to indicate a metabolic slowdown associated with MCFA oxidation at reperfusion. This metabolic slowdown may prove beneficial in preserving mitochondrial function and promoting contractile recovery for the ucp3^+/−^ hearts. There is mounting evidence that rapid recovery of mitochondrial respiration in the post-ischemic period promotes damage to the oxidative phosphorylation machinery that impedes full recovery of cardiac function. For example, high glucose utilization during reperfusion can accelerate electron transfer through a respiratory chain that has sustained oxidative damage, a situation that increases the production of superoxide and downstream ROS [16]. In healthy hearts, the use of respiratory chain inhibitors to block electron transport during ischemia and reperfusion has proven to be cardioprotective [4, 21]. Reversible inhibition of complex I activity limits oxyradical production by decreasing electron flow into complex III and by decreasing reverse electron transport from complex II to complex I [20, 50]. In this case, the wash-out of inhibitors at reperfusion is believed to afford protection by facilitating a more gradual recovery of mitochondrial function, thereby avoiding a burst of ROS and Ca^2+^ overload in mitochondria [18]. Since a high membrane potential can contribute to ROS production, the activation of UCPs during increased rates of electron transfer serves a physiological role by slightly decreasing the membrane potential [13, 14]. Thus, we propose that MCFAs may compensate for a partial lack of myocardial UCP3 at reperfusion by preventing excessive mitochondrial respiration and ROS-mediated mitochondrial dysfunction (Fig. 8).

In conclusion, our results demonstrate that normal UCP3 protein levels are a critical determinant of optimal recovery of mitochondrial LCFA oxidation and contractility in the post-ischemic heart. Moreover, our findings reveal a potential mechanism for the increased sensitivity of obese, insulin-resistant type 2 diabetic individuals toward I/R injury. We acknowledge that the main energetic problem for the diabetic heart has historically been attributed to the impairment of glucose oxidation [1, 47, 48]. Although therapies promoting glucose oxidation (e.g. GIK, rosiglitazone) can improve functional recovery of the diabetic heart after ischemia [29, 35], adverse effects (hyperglycemia, cardiovascular events) may outweigh clinical benefits of such strategies [22, 40, 52]. Matching fatty acid metabolism to the energetic needs of the heart has been proposed as an alternative intervention to preserve cardiac function in pathological situations [39]. We propose that reperfusion with MCFAs may be an effective metabolic intervention for improving the recovery of the type 2 diabetic heart following ischemia.

## Electronic supplementary material

Below is the link to the electronic supplementary material.

Supplementary material 1 (PDF 425 kb)
