# Supplementary material for: Uncoupling protein 3 deficiency impairs myocardial fatty acid oxidation and contractile recovery following ischemia/reperfusion
Source: Basic Res Cardiol. 2018 Oct 29;113(6):47. doi: 10.1007/s00395-018-0707-9 (PMC6208686; doi:10.1007/s00395-018-0707-9)

Uncoupling Protein 3 Deficiency Impairs Myocardial Fatty Acid Oxidation and Contractile Recovery Following Ischemia/Reperfusion

Kristin S. Edwards[1, 2, 3], Sadia Ashraf[1, 2, 3], Tyler M. Lomax[1, 2, 3], Jessica M. Wiseman[1, 2, 3], Michael E. Hall[1, 2, 3, 4], Fabio N. Gava[1, 2], John E. Hall[1, 2], Jonathan P. Hosler[5] and Romain Harmancey[1, 2, 3]

[1] Department of Physiology and Biophysics, [2] Mississippi Center for Obesity Research, [3] Mississippi Center for Heart Research, [4] Department of Medicine, and [5] Department of Cell and Molecular Biology, University of Mississippi Medical Center, Jackson, MS, USA.

Address for Correspondence:

Romain Harmancey

Department of Physiology and Biophysics,

University of Mississippi Medical Center,

2500 N. State St., Jackson MS 39216-4505, USA

(Phone) +1601-815-0196 (Fax) +1601-984-1817

E-mail: rharmancey@umc.edu

ORCID: 0000-0002-5040-4500

Running Title: UCP3 deficiency and myocardial reperfusion injury

Supplemental Methods

Generation of UCP3-deficient rats. The UCP3-deficient rats were developed on a Sprague Dawley background strain (Taconic) by Horizon Discovery using CRISPR/Cas9 technology. Use of a short guide RNA targeting the sequence TACAGCGGGCTGGTCGCTGG within exon 3 of the *Ucp3* gene (Ensembl Rnor_6.0:1:165489782) generated two independent frameshift mutations with early stop codons: A single nucleotide insertion at position 6890 and a deletion of 22 nucleotides between positions 6873 and 6894 in the genomic sequence (Supplemental Fig. 1a). The two founder lines (hereinafter referred to as 1-bpINS and 22-bpDEL) were used for all subsequent experiments. Both mutational events led to the loss of a restriction site recognized by the restriction endonuclease SfcI, thereby allowing for genotyping by PCR/restriction-fragment length polymorphism (PCR/RFLP) analysis. Briefly, 10 ng of genomic DNA was amplified by PCR using forward primer 5'-GCTCGGTACCATCCTGACTA-3' and reverse primer 5'-TCCTTGACCCACACTCCA-3' with the following steps: An initial denaturation at 95ºC for 5 min, 35 cycles of denaturation at 95ºC for 30 s, primer annealing at 60ºC for 30 s, and extension at 68ºC for 30 s, and a final extension at 68ºC for 5 min. After amplification, the PCR products were incubated for 1 h at 37ºC with 10 units of the restriction endonuclease SfcI (New England Biolabs) and resolved on a 2% agarose gel (Supplemental Fig. 1b).

For each founder line, global knockout rats ($ucp3^{-/-}$), heterozygous knockout rats ($ucp3^{+/-}$), and wild type controls ($ucp3^{+/+}$) were generated by breeding heterozygous knockout animals. Rats from each genotype were born at the expected Mendelian ratio. Immunoblotting confirmed loss of UCP3 in main tissues expressing the protein, such as brown adipose tissue, skeletal muscle, and the heart. There was no noticeable change in expression for UCP1, UCP2, and ANT1, supporting the absence of off-target effects from the guide RNA and suggesting that loss of UCP3 in rats does not lead to any compensatory increase of other mitochondrial uncouplers (Supplemental Fig. 1c). Immunoblotting revealed that mutation of one gene copy led on average to a 70% decrease in UCP3 levels, with decreases among male and female rats ranging between 55% and 82% (Fig. 1d).

Immunoblotting. Tissue samples were homogenized with a Bio-Gen PRO200 Homogenizer in protein lysis buffer containing 2.5 mM EGTA, 2.5 mM EDTA, 20 mM KCl, 40 mM β-glycerophosphate, 40 mM NaF, 4 mM NaPPi, 10% glycerol, 0.1% Nonidet-P40, and cOmplete Protease Inhibitor Cocktail (Roche). Tissue homogenates were subsequently centrifuged at 13,000 rpm for 5 min at 4ºC and protein concentration of the supernatant determined by bicinchoninic acid assay. Proteins were separated by polyacrylamide gel electrophoresis and transferred to 0.45 µm pore size polyvinylidene difluoride membranes. Membranes were blocked for 1 hour with 5% milk in 1X Tris-buffered saline, 0.4% Tween 20 at room temperature and incubated overnight at 4ºC with primary antibodies against UCP1 + UCP3 (Abcam ab193470), UCP2 (Cell Signaling Technology #89326), adenine nucleotide translocase 1 (ANT1; Abcam ab102032), heat shock protein 60 (HSP60; Cell Signaling Technology #12165), or voltage-dependent anion channel (VDAC; Cell Signaling Technology #4661). Protein detection was carried out using horseradish peroxidase (HRP)-conjugated anti-rabbit IgG and chemiluminescence. Densitometric analyses were performed with ImageJ 1.48v.

Working heart preparation. Perfusions were carried out on spontaneously beating hearts with a filling pressure of 15 cmH_2O and an afterload of 100 cmH_2O, using nonrecirculating Krebs-Henseleit buffer equilibrated with 95% O_2-5% CO_2 and warmed at 37ºC as the perfusate. The perfusate [Ca^{2+}] was 2.5 mM and was supplemented with 5.5 mM glucose, 0.8 mM fatty acids bound at a 5.3:1 (mole:mole) ratio to bovine serum albumin (BSA), fatty acid-free (Probumin; EMD Millipore), and insulin (40 µU/ml). Depending on the experimental protocol, fatty acids consisted either of the LCFA oleate, the MCFA octanoate, or an equimolar solution of both fatty acids together. Myocardial rates of glucose and fatty acid oxidation were determined by quantitative collection of [^{14}C]O_2 and [^{3}H]$_2O$ released in the coronary effluent using [U-^{14}C]glucose (0.08 µCi/ml), [9,10-^{3}H]oleate (0.1 µCi/ml), and *n*-[8-^{3}H]octanoate (0.08 µCi/ml) as the radiolabeled tracers. Aortic flow and coronary flow were measured every 5 min. Buffer O_2 concentration was recorded continuously with MI-730 Oxygen Electrodes (Microelectrodes, Inc.) connected to a PowerLab 8/35 data acquisition system with LabChart 8 software (ADInstruments). Cardiac power (milliwatts) was calculated as the product of cardiac output (coronary plus aortic flow, m^3/s) and the afterload value (pascals). Myocardial oxygen consumption (MV_{O2}; µmol/min) was determined using 1.06 mM for the concentration of dissolved O_2 at 100% saturation [7]. All cardiac parameters were normalized per gram of dry tissue weight (gdw).

Determination of mitochondrial function. Heart mitochondria were analyzed with an Oxygraph-2k multi-sensor system (O2k, Oroboros Instruments). Respiration driven by non-fatty acid substrates was measured at 37ºC in FAO buffer (10 mM KH_2PO_4, 5 mM $MgCl_2$, 30 mM KCl, 1 mM EDTA, 75 mM Tris, pH 7.5) by adding, in this order, 60-80 µg of intact mitochondria, respiratory substrates, and 2 mM ADP. The respiratory control ratio (RCR) was calculated as the ratio between ADP-stimulated (state 3) and resting (state 2) respiration rates. Mitochondrial oxidation of free fatty acids was determined essentially by the method of Groot and Hulsmann [2]. Briefly, FAO buffer was supplemented with 2 mM ATP, 1 mM NAD^+, 25 µM cytochrome *c*, 0.1 mM coenzyme A and 0.5 mM malate. Oxygen consumption was initiated by the addition of 0.5 mM fatty acid previously bound at a 7:1 (mole:mole) ratio to fatty acid-free BSA. The oxidation of oleate also required 2 mM L-carnitine to allow for its import by the carnitine shuttle. Carnitine was not required for the import of octanoate. Mitochondrial membrane potential or hydrogen peroxide generation were measured with Safranin O and Amplex Red, respectively [3, 4].

Respiratory complex activities. Respiratory complexes were analyzed as previously described [1, 5], all at 25°C in 0.5 ml reactions. In brief, broken mitochondria were generated by resuspension of intact mitochondria in 50 mM Tris buffer, pH 7.4, 39.6 mM KCl (TBK), followed by two freeze-thaw cycles with liquid nitrogen. Complex I (NADH dehydrogenase) activity was measured as the oxidation of NADH at 340 nm ($\varepsilon = 6.22\ mM^{-1}\ cm^{-1}$). The assay mixture consisted of TBK, 9 µM antimycin A, 100 µM ubiquinone 1 (UQ1), 3 mM KCN and 60-80 µg of broken mitochondria. The reaction was started by adding 100 µM NADH. After obtaining the initial rate, rotenone was added to 5 µM. The inhibited rate was subtracted to obtain the rate of rotenone-sensitive NADH oxidation. Complex III (cytochrome *bc*1 complex) activity was measured by monitoring the reduction of horse heart cytochrome *c* at 550 nm ($\varepsilon = 28.5\ mM^{-1}\ cm^{-1}$). The assay mixture consisted of TBK, 4 µM rotenone, 0.1 mM EDTA, 60 µM cytochrome *c*, 3 mM KCN and 60-80 µg broken mitochondria. The reaction was initiated by the addition of 100

μM decylubiquinol (DQH_2). Complex IV (cytochrome *c* oxidase) activity was measured as O_2 consumption using the Oroboros O2k. TBK was supplemented with 3 mM ascorbate, 0.3 mM N,N,N′,N′-tetramethyl-p-phenylenediamine (TMPD), 40 μM horse heart cytochrome *c*, 5 μM myxothiazol, and 5 μM antimycin A. The reaction was initiated with 60-80 µg broken mitochondria.

Carnitine palmitoyl transferase activity assay. Carnitine palmitoyl transferase (CPT) activity was determined using the isotope forward assay, which measures the rate of oleoylcarnitine formation when oleoyl-CoA and labeled carnitine are incubated with a tissue homogenate [8, 9]. Briefly, frozen cardiac tissue samples were homogenized in a solution containing 50 mM Tris Buffer, pH 7.6, 100 mM KCl, 5 mM $MgSO_4$, 1 mM EDTA, and 1 mM ATP. The standard reaction system contained 0.1 M Tris buffer, pH 7.6, 2 mM KCN, 0.1% fatty acid-free BSA, 1 mM dithiothreitol, 0.08 mM oleoyl-CoA, and 5 mM L-Carnitine with 0.04 µCi L-[N-methyl-^{14}C]carnitine/µmol in a final volume of 1.0 ml. The reaction was started by the addition of 50 µg of tissue protein lysate and continued for 10 min at 30ºC. The reaction was stopped and the reaction product [14C]oleoylcarnitine was extracted by the method of Solberg [6] using isobutanol and ammonium sulfate saturated water, and 0.5 ml of the organic phase was assayed for radioactivity in a liquid scintillation counter.

Supplemental References

1. Barrientos A (2002) In vivo and in organello assessment of OXPHOS activities. Methods 26:307-316 doi:10.1016/S1046-2023(02)00036-1
2. Groot PH, Hulsmann WC (1973) The activation and oxidation of octanoate and palmitate by rat skeletal muscle mitochondria. Biochim Biophys Acta 316:124-135 doi:10.1016/0005-2760(73)90002-7
3. Krumschnabel G, Eigentler A, Fasching M, Gnaiger E (2014) Use of safranin for the assessment of mitochondrial membrane potential by high-resolution respirometry and fluorometry. Methods Enzymol 542:163-181 doi:10.1016/B978-0-12-416618-9.00009-1
4. Krumschnabel G, Fontana-Ayoub M, Sumbalova Z, Heidler J, Gauper K, Fasching M, Gnaiger E (2015) Simultaneous high-resolution measurement of mitochondrial respiration and hydrogen peroxide production. Methods Mol Biol 1264:245-261 doi:10.1007/978-1-4939-2257-4_22
5. Shirey K, Stover KR, Cleary J, Hoang N, Hosler J (2016) Membrane-Anchored Cyclic Peptides as Effectors of Mitochondrial Oxidative Phosphorylation. Biochemistry 55:2100-2111 doi:10.1021/acs.biochem.5b01368
6. Solberg HE (1972) Different carnitine acyltransferases in calf liver. Biochim Biophys Acta 280:422-433 doi:10.1016/0005-2760(72)90248-2
7. Starnes JW, Wilson DF, Erecinska M (1985) Substrate dependence of metabolic state and coronary flow in perfused rat heart. Am J Physiol 249:H799-806 doi:10.1152/ajpheart.1985.249.4.H799
8. Zierz S, Engel AG (1987) Different sites of inhibition of carnitine palmitoyltransferase by malonyl-CoA, and by acetyl-CoA and CoA, in human skeletal muscle. Biochem J 245:205-209 doi:10.1042/bj2450205
9. Zierz S, Engel AG (1985) Regulatory properties of a mutant carnitine palmitoyltransferase in human skeletal muscle. Eur J Biochem 149:207-214 doi:10.1111/j.1432-1033.1985.tb08913.x

Supplemental Table 1 Morphometric and echocardiographic parameters of wild type (+/+) and UCP3-deficient (+/-) rats between 18 and 24 weeks of age.

	Male		Female	
Ucp3	+/+ (n=13)	+/- (n=6)	+/+ (n=4)	+/- (n=4)
BW, g	522.8 ± 80.4	409.8 ± 56.0	327.5 ± 13.0	302.5 ± 41.7
LVM, mg	1393.5 ± 243.9	1403.5 ± 256.3	1105.4 ± 272.8	943.2 ± 228.3
LVM/BW, mg/g	2.7 ± 0.4	3.4 ± 1.2	3.4 ± 0.7	3.3 ± 0.6
HR, bpm	343.2 ± 33.6	365.2 ± 20.0	334.3 ± 16.1	359.6 ± 18.3
LVEDD, mm	8.9 ± 0.9	9.0 ± 0.4	7.7 ± 0.7	7.8 ± 0.8
LVESD, mm	4.9 ± 0.8	5.1 ± 0.5	4.3 ± 0.9	4.5 ± 0.5
LVEDV, µl	444.7 ± 93.1	446.1 ± 47.8	318.7 ± 61.6	332.1 ± 80.7
LVESV, µl	117.4 ± 41.1	123.1 ± 26.9	86.6 ± 39.4	96.0 ± 23.8
LVPWd, mm	2.1 ± 0.2	1.9 ± 0.4	1.9 ± 0.4	1.8 ± 0.2
SV, µl	327.3 ± 54.6	323.0 ± 41.6	232.1 ± 35.5	236.1 ± 57.3
EF, %	74.3 ± 5.0	72.4 ± 5.1	73.7 ± 8.1	71.1 ± 1.3
FS, %	45.4 ± 4.8	43.7 ± 4.6	44.6 ± 6.9	41.8 ± 1.2

BW body weight, EF ejection fraction, FS fractional shortening, HR heart rate, LVEDD left ventricular end diastolic diameter, LVEDV left ventricular end diastolic volume, LVESD left ventricular end systolic diameter, LVESV left ventricular end systolic volume, LVM left ventricular mass, LVPWd left ventricular posterior wall thickness at end diastole, SV stroke volume. Data are presented as mean ± SD.

Supplemental Figure Legends

Supplemental Fig. 1 CRISPR/Cas9-mediated generation of UCP3-deficient rats. **a** Part of exon 3 sequence of the rat UCP3 gene depicting the target site for the single guide RNA (grey box) and the location of a restriction site for the endonuclease SfcI (dotted line). Two founder lines were generated by frameshift mutations caused by a single adenine nucleobase insertion at position 6890 (bold) and a deletion of 22 nucleotides between positions 6873 and 6894 (plain line) in the genomic sequence. **b** Loss of the restriction site recognized by the endonuclease SfcI allows for genotyping by PCR/RFLP analysis. Top black arrow indicates the native (n) PCR product. Bottom two arrows indicate cleaved (c1 and c2) PCR product after successful digestion by SfcI. **c** Immunoblotting confirmation of partial and total loss of UCP3 protein expression in tissues from ucp3$^{+/-}$ and ucp3$^{-/-}$ rats, respectively. The expression levels of other known mitochondrial uncoupling proteins UCP1, UCP2, and ANT1 were not affected by UCP3 deletion. **d** The loss of a single UCP3 gene copy led on average to a 70% decrease in UCP3 protein levels in the heart of ucp3$^{+/-}$ rats. Data are presented as mean $\pm$ SE. *****p < 0.05 vs. ucp3$^{+/+}$. M: Male, F: Female.

Supplemental Figure 1

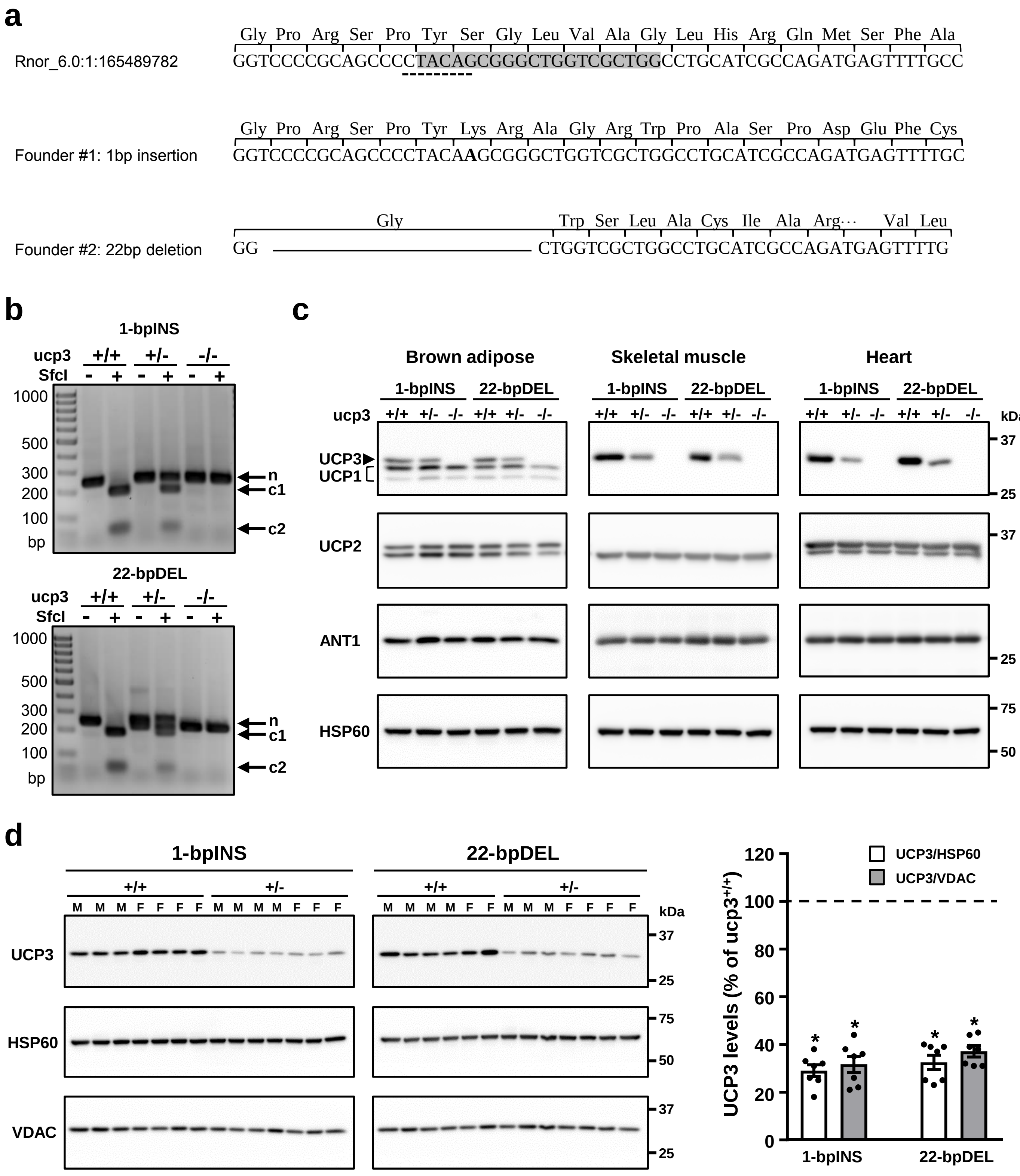

Supplement: Supplementary file 1 — Supplementary material 1 (PDF 425 kb) [file 395_2018_707_MOESM1_ESM.pdf]
